# Characterization of HGF/Met Signaling in Cell Lines Derived From Urothelial Carcinoma of the Bladder

**DOI:** 10.3390/cancers6042313

**Published:** 2014-11-25

**Authors:** Young H. Lee, Andrea B. Apolo, Piyush K. Agarwal, Donald P. Bottaro

**Affiliations:** 1 Urologic Oncology Branch, Center for Cancer Research, National Cancer Institute, National Institutes of Health, Bethesda, MD 20892, USA; E-Mails: leeyh@mail.nih.gov (Y.H.L.); piyush.agarwal@nih.gov (P.K.A.); 2 Genitourinary Malignancies Branch, Center for Cancer Research, National Cancer Institute, National Institutes of Health, Bethesda, MD 20892, USA; E-Mail: andrea.apolo@nih.gov

**Keywords:** bladder cancer, urothelial carcinoma, hepatocyte growth factor, Met, tumor growth, tumor cell invasion

## Abstract

There is mounting evidence of oncogenic hepatocyte growth factor (HGF)/Met signaling in urothelial carcinoma (UC) of the bladder. The effects of three kinase inhibitors, cabozantinib, crizotinib and EMD1214063, on HGF-driven signaling and cell growth, invasion and tumorigenicity were analyzed in cultured UC cell lines. SW780 xenograft growth in SCID and human HGF knock-in SCID (hHGF/SCID) mice treated with cabozantinib or vehicle, as well as tumor levels of Met and pMet, were also determined. Met content was robust in most UC-derived cell lines. Basal pMet content and effector activation state in quiescent cells were low, but significantly enhanced by added HGF, as were cell invasion, proliferation and anchorage independent growth. These HGF-driven effects were reversed by Met inhibitor treatment. Tumor xenograft growth was significantly higher in hHGF/SCID mice *vs.* SCID mice and significantly inhibited by cabozantinib, as was tumor phospho-Met content. These studies indicate the prevalence and functionality of the HGF/Met signaling pathway in UC cells, suggest that paracrine HGF may contribute to UC tumor growth and progression, and that support further preclinical investigation of Met inhibitors for the treatment of UC is warranted.

## 1. Introduction

In 2013, 72,570 new cases of bladder cancer (BCa) and 15,210 bladder cancer-related deaths were estimated in the U.S. alone [1]. Although 70% of newly diagnosed disease is confined to the mucosa, recurrence and progression are frequent and long-term surveillance by cystoscopy is required. The remaining 30% of new cases are more advanced, with muscle-invasion, lymph node involvement or distant metastases. Half of those individuals with muscle-invasive bladder cancer fail definitive therapy (surgery or chemoradiation) within 5 years and succumb to the disease [1,2]. The 5- and 10-year survival rates for patients with lymph node involvement are 31% and 23%, respectively [2]. Standard of care combination platinum-based chemotherapy for patients with metastatic disease provides a median survival of only 15 months and a 5-year survival rate of 15% [3]. BCa has the highest costs per patient in the U.S. compared to all other cancers, reflecting disease prevalence as well as costs of long term monitoring [4]. These circumstances underscore the urgent need to develop new diagnostic and prognostic methods and identify therapeutic targets for this disease.

The cell surface receptor tyrosine kinase (RTK) for hepatocyte growth factor (HGF), known as Met, is widely present in cells of epithelial origin. HGF/Met signaling is frequently implicated in cancer, driving tumor invasiveness and metastasis [5]. Evidence of HGF/Met pathway involvement in BCa has been found in model systems [6,7,8] and *in vivo* [9,10,11]. A recent analysis of 97 high-grade BCa tumors designed to identify actionable drug targets found RTK/RAS/RAF/MAPK pathway alterations in 35% of samples [12]. The RTK genes frequently altered in this cohort were *FGFR3* (13%), *ERBB2* (6%), *FGFR1* (6%) and *MET* (2%) [12]. The RAS/RAF/MAPK cascade is a well-established mediator of HGF/Met driven proliferation and invasiveness [5]. A preliminary interrogation of the four BCa datasets in The Cancer Genome Atlas (TCGA) project using the cBioPortal [13,14] further revealed HGF upregulation (>2-fold) in 7% of cases in the same dataset used by Iyer *et al.* [12], as well as mutually exclusive oncogenic alterations (amplification, overexpression or mutation) in *MET* or *HGF* genes in 3% and 2%, respectively, of cases in the Nature Genetics 2013 dataset (*n =* 99) [15], in 10% and 4%, respectively, of cases in the Nature 2014 dataset (*n =* 131) [16], and in 8% and 3% of cases, respectively, in the TCGA Provisional dataset (*n =* 201). Together these studies identified genetic aberrations in *HGF* and *MET* at an average combined frequency of 10% in 528 BCa cases.

While several Met kinase inhibitors are in preclinical and clinical development, there use for the treatment of BCa has been limited. Here, we focused on three small molecule Met inhibitors, crizotinib, cabozantinib and EMD1214063. Crizotinib is an orally bioavailable small molecule inhibitor of Met and anaplastic lymphoma kinase (ALK), which has been US FDA approved for the treatment of ALK-positive non-small cell lung cancer [17]. Crizotinib also less potently inhibits Ron and Axl, and blocks cell proliferation, invasion and survival in numerous tumor cell types, including those derived from head and neck carcinoma, gastric cancer, glioblastoma, and breast cancer [18,19,20]. Cabozantinib is a potent inhibitor of several tyrosine kinase receptors including Met, VEGFR2, Axl, Flt3, Kit, Tie2 and Ret [21,22]. Preclinical studies show that cabozantinib can block tumor growth and invasion in breast, lung, prostate, and pancreatic cancer derived cells [21,23,24]; cabozantinib has been US FDA approved for the treatment of advanced medullary thyroid carcinoma [17]. EDM1214063 is a Met selective inhibitor with no other known kinase targets which has induced regression of human gastric, lung, and pancreatic tumor xenografts in mice [25].

Accurately selecting therapies that will be effective against a specific tumor is the ultimate goal of clinicians treating BCa. In a prior study we found that levels of soluble Met ectodomain (sMet), which are readily measured in the urine of bladder cancer patients and those with no evidence of cancer, could distinguish patients with BCa from those without, and patients with or without muscle-invasive BCa, suggesting the potential utility of urinary sMet as a BCa biomarker for surveillance following initial treatment [26]. In parallel with our continued analysis of HGF/Met signaling human BCa tissue samples, the present preclinical study was designed to interrogate potentially oncogenic HGF/Met signaling in a collection of UC derived cell lines to better define the nature and extent of HGF/Met pathway involvement in BCa. Our results indicate that paracrine HGF/Met signaling is capable of driving growth, invasion and tumorigenesis in UC derived cells; these findings lay the foundation for assessing pathway status in patient samples to identify those most likely to benefit from HGF/Met pathway targeted therapies.

## 2. Results

**Figure 1:**
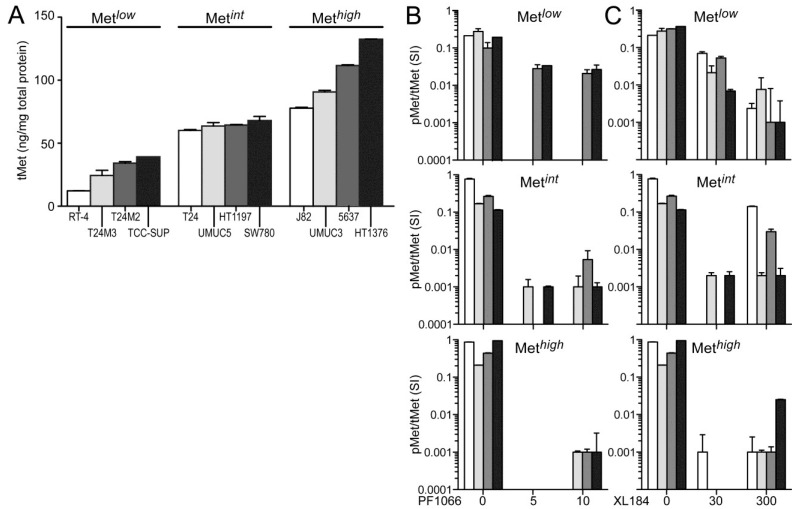
Total and phospho-Met content in UC-derived cell lines. (**A**) Cell types indicated were serum-deprived overnight before extraction with non-ionic detergent and measurement of total Met protein abundance by electrochemiluminescent immunoassay. The 12 cell lines were divided into three groups by Met abundance: low (Met*^low^*), intermediate (Met*^int^*) and high (Met*^high^*). Met abundance in each subset is further indicated by bar grayscale intensity from least (white) to most (black); this bar color scheme is in all figures except 6D; (**B**,**C**) Serum-deprived cells were treated with (**B**) 5 nM or 10 nM of crizotinib (PF1066) or (**C**) 30 nM or 300 nM of cabozantinib (XL184) for 20 min, followed by concurrent 20 min exposure to HGF (1 nM). Phospho-Met (pMet) in cell lysates was measured by two-site electrochemiluminescent immunoassay. Y-axis in log_10_; bars represent the mean of triplicate samples ± standard deviation (SD) and are representative of three independent experiments. Some error bars are too small to be visible.

The absolute cellular Met protein content values (normalized to total extracted cell protein) for multiple human tumor derived cell lines (including colon, skin, breast, kidney, prostate, lung, and gastric cancer) and 12 UC derived cell lines as measured by electrochemiluminescent two-site immunoassay are listed in Table 1. The highest Met protein level was found in the MET amplified gastric carcinoma derived cell line MKN45 (800 pg Met/µg total protein), and the lowest was found in the human breast adenocarcinoma derived cell line MCF7 (1 pg Met/µg total protein; Table 1). Met content among UC derived cell lines was comparable or greater than that found in many other HGF-responsive tumor-derived cell lines. RT4, derived from a Ta stage bladder tumor, had the lowest Met level among the UC cell lines studied here. All cell lines derived from higher stage tumors, TCC-SUP, T24, T24M2, T24M3, HT1197, SW780, J82, UMUC3, UMUC5, 5637, and HT1376 cells, exhibited higher Met content than RT4 (Figure 1A). In the interest of detecting differences in HGF-responsiveness, or sensitivity to Met inhibition, that might correlate with Met abundance without exhaustively testing all 12 cell lines in all cell-based assays used, we organized the cell lines into three groups of 4 with low (Met*^low^*), intermediate (Met*^int^*) or high (Met*^high^*) Met content (Figure 1A). This organization was used in the presentation of all cell-based assay results.

None of the UC derived cell lines produced detectable levels of HGF, as determined by two-site electrochemiluminescent immunoassay (200 femtomole limit of detection), suggesting that autocrine HGF/Met signaling is rare in UC of the bladder. Treatment with exogenous HGF (1 nM) induced significant Met kinase activation (pMet/Met SI ratio) in all UC derived cell lines within 20 min, which was suppressed by the small molecule Met kinase inhibitors crizotinib at 5 and 10 nM, (corresponding to IC_50_ and IC_90_ values, respectively; Figure 1B) and cabozantinib at 30 and 300 nM (corresponding to IC_50_ and IC_90_ values, respectively; Figure 1C), for which Met is the only common high affinity target. Neither inhibitor affected Met abundance at any concentration tested for time periods up to 1 week.

**Table 1:** Met protein content (pg/ug total protein) in selected tumor derived cell lines.

Cell Line	Description	Mean Met ± SD (pg/µg)
**colon**		
SW480	Dukes’ type B colorectal adenocarcinoma, Ras mutation	3 ± 0.09
SW620	lymph node metastasis of colorectal cancer	120 ± 5.00
**skin**		
CRL7636	skin melanoma	8 ± 0.35
CRL7637	lymph node metastasis of skin melanoma	3 ± 0.10
**breast**		
HTB 125	normal mammary epithelium	13 ± 0.34
MCF7	human breast adenocarcinoma cell line	1 ± 0.02
**kidney**		
786-0	clear cell RCC, VHL negative	45 ± 1.12
ACHN	papillary RCC, Met T1010I mutation	49 ± 3.00
**prostate**		
PC3	prostate carcinoma	47 ± 2.02
PC3M	metastatic prostate carcinoma, MxA negative	83 ± 2.77
**lung**		
H596	human lung adenosquamous carcinoma	9 ± 0.08
A549	human lung adenocarcinoma epithelial cell line	66 ± 3.95
**gastric**		
MKN45	human gastric carcinoma, MET amplified	800 ± 45
**bladder**		
RT4	human bladder epithelial	12 ± 0.10
TCCSUP	urinary bladder anaplastic transitional	39 ± 0.00
T24	urinary bladder transitional	60 ± 0.70
T24M2	metastatic human bladder epithelial	34 ± 1.30
T24M3	metastatic human bladder epithelial	24 ± 4.10
J82	urinary bladder transitional	76 ± 0.10
SW780	urinary bladder transitional	109 ± 4.82
UMUC3	urinary bladder transitional	91 ± 1.30
UMUC5	urinary bladder transitional	64 ± 2.76
5637	urinary bladder grade II carcinoma	112 ± 0.61
HT1197	urinary bladder grade IV carcinoma	64 ± 0.50
HT1376	urinary bladder grade III carcinoma	133 ± 0.20

**Figure 2:**
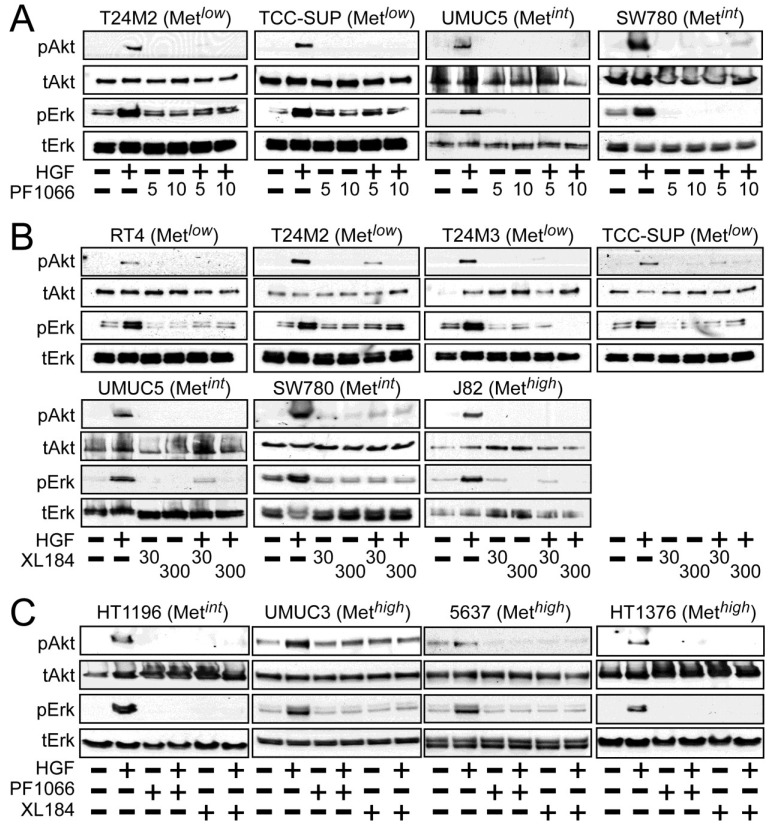
Crizotinib and cabozantinib inhibit HGF/Met-mediated PI3K and MAPK pathway activation. Cell types indicated were treated with (**A**) 5 nM or 10 nM of crizotinib (PF1066); or (**B**) 30 nM or 300 nM of cabozantinib (XL184) for 20 min, followed by concurrent 20 min treatment with HGF (1 nM); or (**C**) 10 nM crizotinib or 300 nM cabozantinib, as indicated. Cell lysates were then extracted with non-ionic detergent, separated by SDS-PAGE and immunoblotted for pAkt, Akt, pErk, or Erk as indicated.

Activation of the phosphotidylinositol 3-kinase (PI3K) and MAPK pathways downstream of Met mediates HGF-driven cell survival, migration, invasion and proliferation. HGF stimulated both Akt and Erk phosphorylation in all UC derived cell lines (Figure 2). Treatment with 5 and 10 nM crizotinib suppressed HGF-activated pErk and pAkt in the Met*^low^* lines T24M2 and TCC-SUP (Figure 2A, left), the Met*^int^* lines UMUC5 and SW780 (Figure 2A, right) and the Met*^high^* lines UMUC3, 5637 and HT1376 (Figure 2C). Treatment with cabozantinib at 30 and 300 nM also uniformly inhibited HGF-induced Erk and Akt activation in the Met*^low^* lines RT4, T24M2, T24M3, and TCC-SUP (Figure 2B, top), the Met*^int^* lines UMUC5, SW780 (Figure 2B, bottom) and HT1196 (Figure 2C, left), and the Met*^high^* lines J82 (Figure 2B, bottom), UMUC3, 5637 and HT1376 (Figure 2C). These findings show that HGF-stimulated activation of Akt and Erk could be inhibited by either drug in all UC cell lines tested, regardless of Met content.

**Figure 3:**
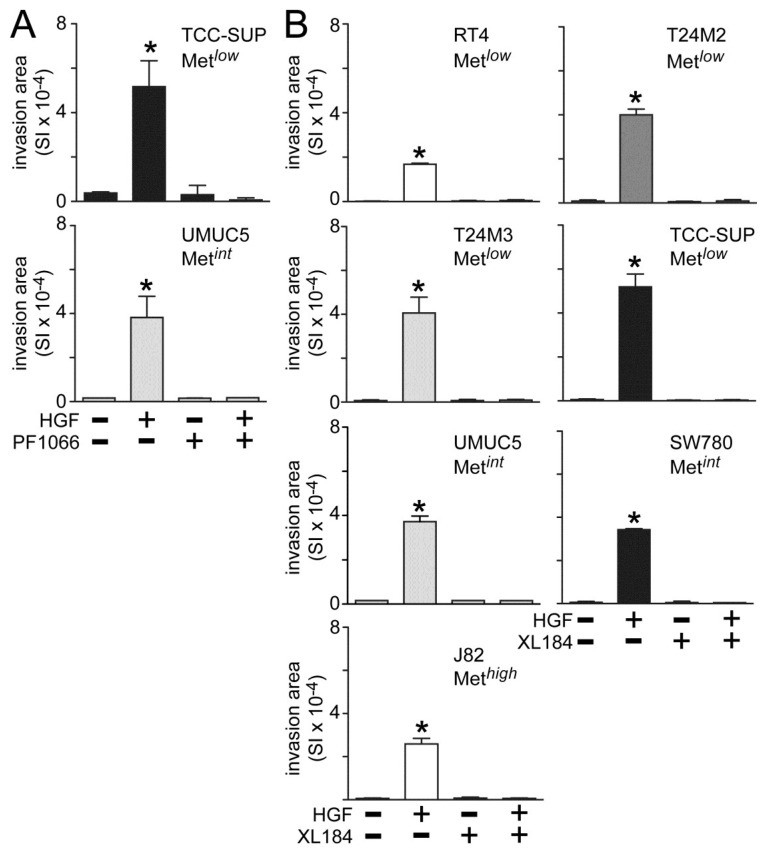
Crizotinib and cabozantinib inhibit HGF-driven UC cell invasion. Cell invasion was measured in matrigel-coated Boyden chambers. Cell types indicated were treated with (**A**) 5 nM of crizotinib (PF1066) or (**B**) 30 nM of cabozantinib (XL184) in the absence or the presence of 1 nM HGF for 24 h. Bars represent the mean of triplicate samples ± SD. Data are representative of three independent experiments; asterisks (*) indicate statistically significant differences from the control group (*p <* 0.05). Some error bars are too small to be visible.

Both crizotininb and cabozantinib also suppressed HGF-induced UC cell invasion across matrigel-coated Boyden chambers. Invasion was significantly increased by HGF treatment in all cell lines tested (Figure 3). Concomitant crizotinib treatment (5 nM) suppressed HGF-driven invasion by the Metlow line TCC-SUP and the Metint line UMUC5 (Figure 3A). Concomitant cabozantinib treatment (30 nM) also suppressed invasion by the Met*^low^* lines RT4, T24M2, T24M3 and TCC-SUP, the Met*^int^* lines UMUC5 and SW780, and the Met*^high^* line J82 (Figure 3B).

**Figure 4:**
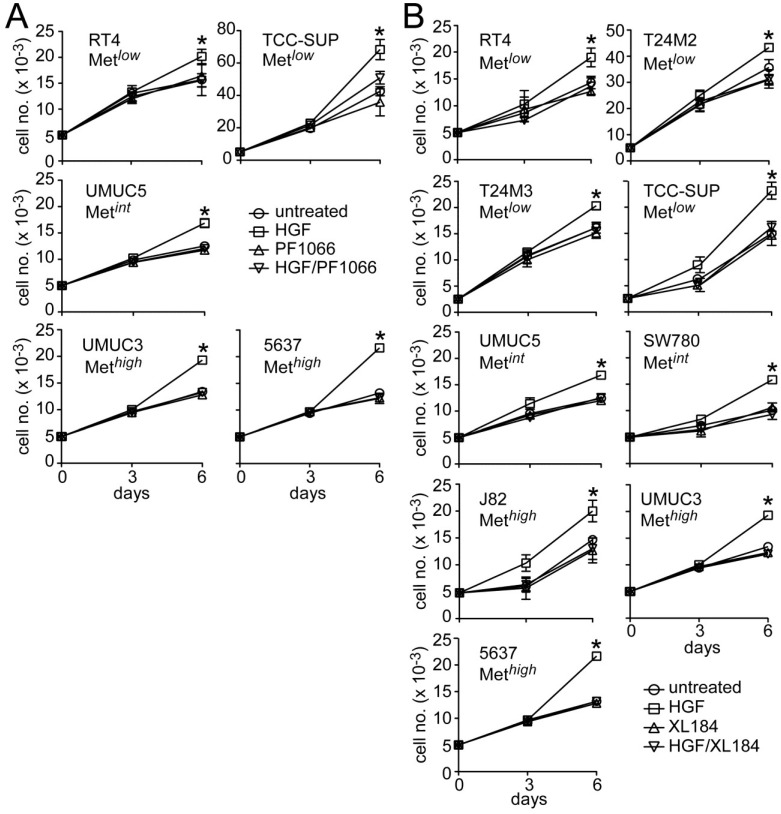
Crizotinib and cabozantinib block HGF-mediated UC cell proliferation. (**A**) Cell growth (mean cell number ± SD) for control (circles) and cells treated with 5 nM crizotinib (PF1066, triangles), 1 nM HGF (squares) or HGF + crizotinib (inverted triangles) on days 0, 1 and 4; (**B**) Cell growth for control (circles) and cells treated with 30 nM cabozantinib (XL184, triangles), 1 nM HGF (squares) or HGF + cabozantinib (inverted triangles) on days 0, 1 and 4. Data are representative of three experiments; asterisks (*) indicate statistically significant differences from the control group (*p <* 0.05).

HGF significantly stimulated growth in all UC derived cell lines tested (Figure 4). Concomitant crizotinib treatment (5 nM) suppressed HGF-driven proliferation by the Met*^low^* lines RT4 and TCC-SUP, the Met*^int^* line UMUC5, and the Met*^high^* lines UMUC3 and 5637 (Figure 4A). Concomitant cabozantinib treatment (30 nM) also suppressed invasion by the Met*^low^* lines RT4, T24M2, T24M3 and TCC-SUP, the Met*^int^* lines UMUC5 and SW780, and the Met*^high^* lines J82, UMUC3 and 5637 (Figure 4B). Growth suppression by these agents was not accompanied by obvious cell death or DNA fragmentation, suggesting that Met TK inhibition blocked cell cycle progression.

**Figure 5:**
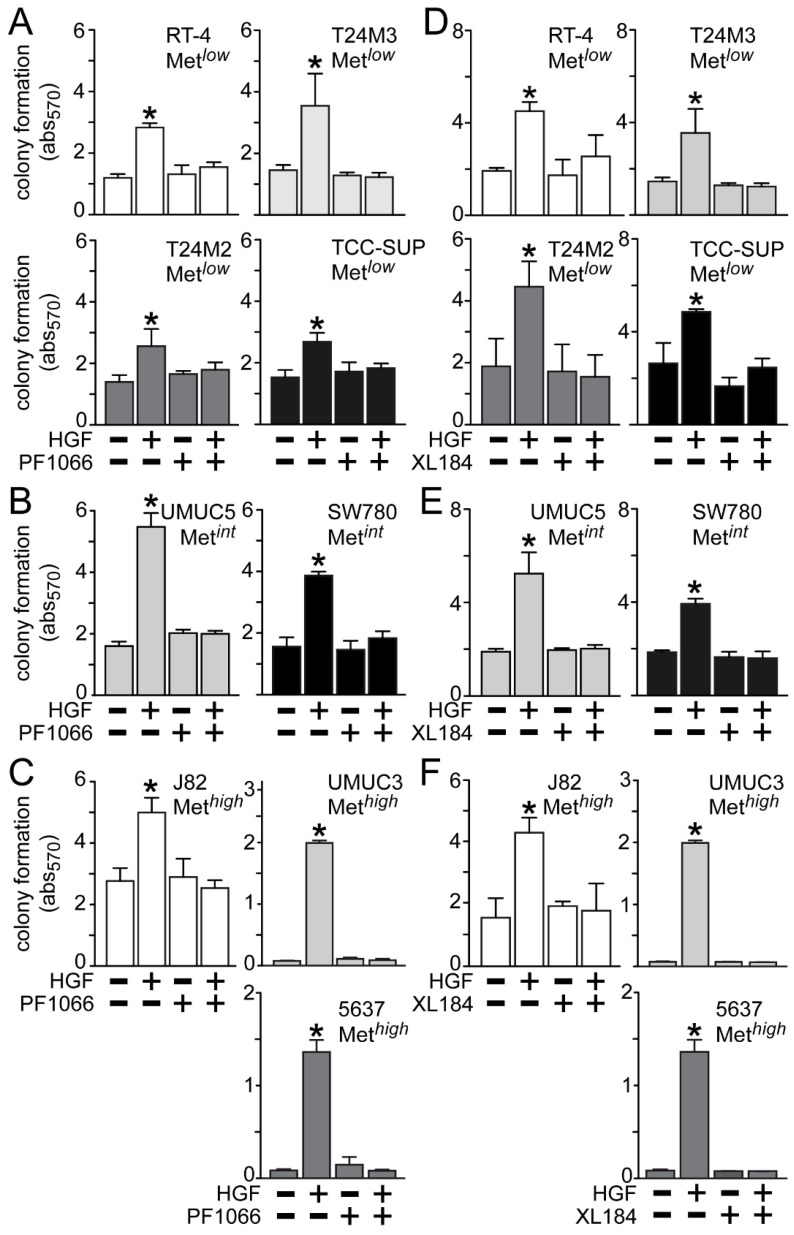
Blockade of HGF/Met-driven anchorage-independent growth by crizotinib and cabozantinib. Cells were plated in soft agar, followed by (**A**–**C**) 5 nM of crizotinib (PF1066) or (**D**–**F**) 30 nM of cabozantinib (XL184) treatment in the absence or the presence of HGF (1 nM) every other day. On day 7 colony growth was measured using MTT. Values represent the mean ± SD of triplicate samples and are representative of three experiments; asterisks (*) indicate significant differences from control (*p <* 0.05).

HGF treatment also significantly stimulated anchorage-independent cell growth (Figure 5), which was suppressed by crizotinib in the Met*^low^* lines RT4, T24M3, T24M2 and TCC-SUP (Figure 5A), the Met*^int^* lines UMUC5 and SW780 (Figure 5B), and the Met*^high^* lines J82, UMUC3 and 5637 (Figure 5C). Similarly, cabozantinib treatment inhibited HGF-driven colony formation in the Met*^low^* lines RT4, T24M3, T24M2 and TCC-SUP (Figure 5D), the Met*^int^* lines UMUC5 and SW780 (Figure 5E), and the Met*^high^* lines J82, UMUC3 and 5637 (Figure 5F).

**Figure 6:**
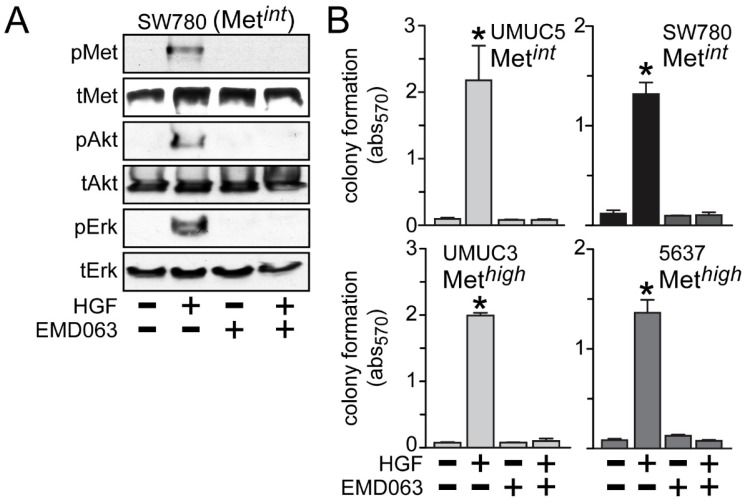
Met inhibition suppresses anchorage-independent soft agar growth. (**A**) SW780 cells were treated with 5 nM of EMD1214063 (EMD063) for 20 min, followed by concurrent treatment with 1 nM HGF for an additional 20 min. Cell lysates were used for SDS-PAGE and immunoblot analysis of pMet, tMet, pAkt, tAkt, pErk and tErk; (**B**) Cell types indicated were embedded in soft agar, followed by 5 nM EMD1214063 (EMD063) treatment in the absence or the presence of HGF (1 nM) every other day. On day 7, MTT was used to quantitate viable cell colonies by absorbance at 570 nm. Values represent the mean ± SD of triplicate samples. Data are representative of at least three independent experiments; * indicates *p <* 0.05 *vs.* control.

Inhibition of anchorage-independent growth by crizotinib or cabozantinib was likely to be solely due to HGF/Met pathway inhibition, since the Met-selective compound EMD1214063 also prevented activation of Met, Akt and Erk by HGF in the Met*^int^* line SW780 (Figure 6A) and suppressed HGF-driven anchorage-independent cell growth by the Met*^int^* lines UMUC5 and SW780, and the Met*^high^* lines UMUC3 and 5637 (Figure 6B).

**Figure 7:**
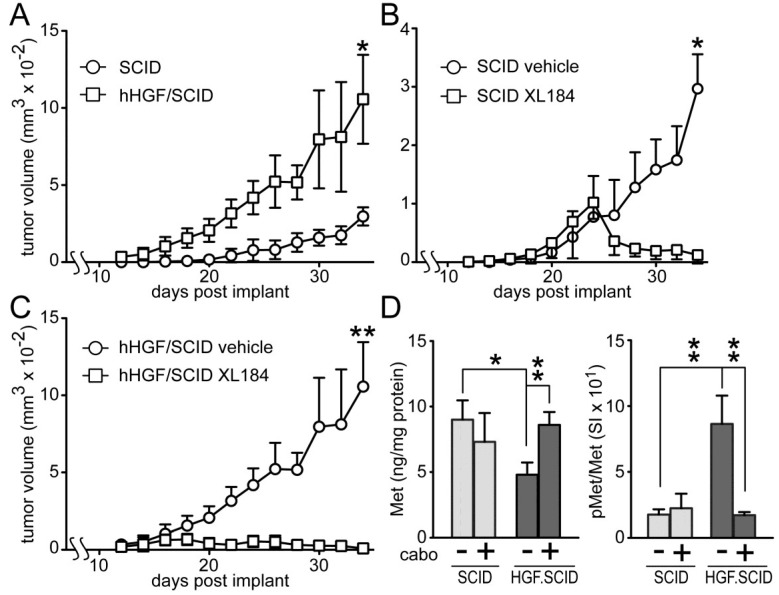
Cabozantinib inhibits SW780 xenograft tumor growth in SCID and hHGF/SCID mice. (**A**) SW780 cells were implanted subcutaneously on both flanks of SCID and hHGF/SCID mice (5 mice per group) and tumor growth over time was measured using calipers. Values represent the mean tumor volume ± SD, *n* = 10 tumors/group. Single asterisk indicates *p <* 0.05 *vs.* vehicle control group; (**B**,**C**) SW780 cells were implanted into both flanks of (**B**) SCID or (**C**) hHGF/SCID mice (5 mice per group). When tumor volume reached 150 mm^3^, mice were treated daily with either vehicle (circles) or cabozantinib (squares). Values represent mean tumor volume ± SD, *n* = 10 tumors/group, * indicates *p <* 0.05, ** indicates *p <* 0.001 *vs.* vehicle control; (**D**) Xenograft tumors from SCID (light gray bars) or hHGF/SCID (dark gray bars) mice from the time course in (**A**) through (**C**) were excised and processed by physical disruption and non-ionic detergent extraction; extracts were clarified by centrifugation and analyzed for total and pMet content. Bars represent the mean ± SD of triplicate samples from 5 tumors/group. Asterisks indicate significant differences at the *p <* 0.05 (*) and *p <* 0.001 (**) levels between bracketed groups.

Cabozantinib suppressed HGF-dependent and HGF-independent UC cell xenograft growth in SCID mice (Figure 7). Murine HGF binds to, but does not fully activate human Met [27]; to circumvent this obstacle to mimicking paracrine HGF driven tumor growth in humans, we used SCID mice that are genetically engineered to express only human HGF (hHGF; hHGF^ki/ki,SCID/SCID^, also known as Hgftm1.1 (HGF)Aveo). Growth of SW780 cell xenografts in SCID mice was significantly slower than in hHGF/SCID mice (Figure 7A), indicating a profound impact of hHGF on tumor growth. Daily administration of cabozantinib by oral gavage (33 mg/kg) after tumors reached 150 mm^3^ (~day 18), significantly blocked, and later reversed, SW780 tumor growth in both SCID (Figure 7B) and hHGF/SCID (Figure 7C) mouse models relative to vehicle treated mice, although the magnitude of this effect was substantially greater in the hHGF/SCID mice. SW780 xenograft tumors excised from the SCID and hHGF/SCID mice showed significant phospho-Met (pMet) elevation in vehicle-treated hHGF/SCID mice and complete suppression of this pMet increase in hHGF/SCID mice treated with cabozantinib (Figure 7D, right). Total tumor Met content was significantly reduced in vehicle-treated hHGF/SCID mice but not vehicle-treated SCID mice, also suggestive of a paracrine hHGF effect (Figure 7D, left).

## 3. Discussion

The HGF/Met pathway was characterized in several UC-derived cell lines to better define its prevalence and functionality in bladder cancer. Our findings that UC cell lines derived from high grade tumors displayed abundant Met protein but no detectable HGF are consistent with the four TCGA bladder cancer datasets which show frequent but mutually exclusive (odds ratio: <0.000001, strong tendency toward mutual exclusivity) overexpression of *HGF* or *MET* genes [13,14], and suggest that this pathway may be activated in a predominantly paracrine mode in bladder cancer. Stromal cells in the bladder wall, vascular endothelial and mural cells, and immune cells are all potential local sources of HGF in the bladder. Plasma HGF is another source, which can fluctuate considerably in cancer. Of these sources, a report on immunohistochemical HGF staining found it undetectable in normal bladder urothelium, but readily observed in a majority of specimens obtained from BCa patients with a range of disease severity [28]. Epithelial localization of HGF was observed in papillary tumors suggestive of autocrine signaling, whereas immunoreactivity in nodular urothelial carcinoma was observed primarily in mesenchymal cells, including fibroblast-like cells and bladder wall smooth muscle cells, consistent with paracrine HGF delivery [28]. Reinforcing the latter, HGF production by a lung-derived fibroblast cell line was significantly enhanced by co-culture with five bladder cancer-derived cell lines, four of which (J82, T24, 5637 and HT1376) were also analyzed in the present report [29]. HGF protein content in bladder tissue extracts and urine were found to correlate significantly with tumor stage and grade [30]. A prior study of 5 rat urothelial carcinoma-derived cell lines also found that all expressed Met, whereas only one appeared to express HGF; oddly, the latter cell line was refractory to endogenously added HGF in cell growth and invasion assays [7]. Finally, short variants of the deoxyadenosine tract element located in the HGF promoter associated with enhanced HGF expression were found in 37 of 70 bladder tumor specimens and correlated significantly with higher tumor grade, although the cell of origin was not identified [31]. These studies strongly support paracrine HGF signaling in BCa generally but also suggest that autocrine signaling could occur in specific tumor histologies.

Mutations in *MET* can confer resistance to crizotinib and other Met kinase inhibitors [5]. Consistent with the relatively low frequency of *MET* gene mutation in the four TCGA bladder cancer datasets (11/569 cases or 1.9% combined), the small molecule Met inhibitors crizotininb, cabozantinib and EMD1214063 uniformly and potently suppressed Met and Met-effector activation, and HGF-driven invasion, growth and soft agar colony formation in all UC cell lines tested. Our cell-based assay results do not reveal a relationship between Met abundance and biological response to HGF, or between Met abundance and drug suppression of HGF-induced responses, in the 12 UC-derived cell lines studied. In this regard BCa is not similar to certain other cancers, such as gastric and lung adenocarcinomas, where frequent *MET* gene amplification leads to ligand-independent activation and enhanced sensitivity to Met inhibition [5]. Expression of the primary targets for EMD1214063 (Met) and crizotinib (Met and Alk) in cell lines derived from urothelial carcinoma of the bladder predicts that the predominant therapeutic effects of these agents is likely to be tumor cell stasis and/or death. In contrast, the multiplicity of cabozantinib targets, which are distributed in both tumor and host cells (vascular endothelial cells, immune cells and other cells in the tumor microenvironment), suggests that predicting a cellular basis for potential therapeutic effects will be difficult. Differential therapeutic effects of cabozantinib observed in an NCI clinical trial for advanced BCa [32] are suggestive of effects on both tumor and host cells and warrant further investigation as to their cellular and molecular basis.

Subcutaneously implanted SW780 cells grew significantly faster in hHGF/SCID mice relative to SCID mice, revealing the ability of paracrine delivered HGF to drive UC tumor growth. Cabozantinib fully suppressed SW780 xenograft growth in mice, and prevented the elevated tumor pMet observed in vehicle-treated hHGF knock-in mice. Additional studies will be needed to discern meaningful differences in efficacy among these agents against UC tumor growth, particularly whether the broader target spectrum of cabozantinib and, to a lesser degree, crizotinib, is potentially advantageous, and whether drug effects on the host, such as on tumor-stroma interactions and the tumor vasculature, also contribute to anti-tumor efficacy.

## 4. Experimental

### 4.1. Reagents

Tissue culture media and supplements were obtained from Invitrogen (Carlsbad, CA, USA). Antibodies against pMet (1234/1235), pErk, tErk, pAkt, and tAkt were obtained from Cell Signaling Technology (Danvers, MA, USA). Anti-Met (C-28) was obtained from Santa Cruz Biotechnology (Santa Cruz, CA, USA). Purified recombinant human HGF was obtained from ProSpec (Rehovot, Israel). Crizotinib, cabozantinib and EMD1214063 were obtained from the Open Chemical Repository Collection, Developmental Therapeutics Program, Division of Cancer Treatment and Diagnosis, National Cancer Institute, National Institutes of Health, Bethesda, MD, USA.

### 4.2. Cell Culture

SW480, SW620, CRL 7636, CRL 7637, HTB 125, MCF7, 786-0, ACHN, PC3, UMUC3, UMUC5, H596, A549, MKN45, RT-4, TCC-SUP, T24, HT1197, SW780, J82, 5637 and HT1376 cells were obtained from ATCC (Manassas, VA, USA). PC3M was obtained from MD Anderson Cancer Center, University of Texas (Houston, TX, USA). T24M2 and T24M3 cell lines were derived from metastases of T24 xenografts in mice. Cells were cultured in DMEM medium containing 10% FBS and antibiotic-antimycotic. Cells were grown in 5% CO2 at 37 °C.

### 4.3. SDS-PAGE, Immunoblot Analysis and Two-Site Immunoassays

Cells were washed with cold PBS, extracted in Laemmli buffer, sonicated, heated for 5 min at 95 °C prior to SDS-PAGE and electrophoretic transfer to nitrocellulose. Membranes were blocked with 5% milk in TBST (Tris Buffered Saline, 0.1% Tween 20) for 1 h at 25 °C then incubated 16 h at 4 °C with primary antibody in TBST/0.5% milk. Membranes were washed three times with TBST, incubated with horseradish peroxidase-labeled secondary antibody for 1 h at 25 °C, washed for 3 h with TBS prior to ECL detection (Pierce Biotechnology, Rockford, lL, USA). Phospho- and total Met content in Triton X-100 cell extracts were analyzed by electrochemiluminescent two site immunoassay using a SectorImager 2400 plate reader (Meso Scale Discovery, Gaithersburg, MD, USA) as described [33]. The assay has attomole sensitivity for total Met; pMet was expressed as signal intensity (SI) normalized to Met/total protein.

### 4.4. Cell Invasion and Proliferation Assays

Invasion assays were performed using 8 um pore size BD Bioscience BioCoat Matrigel Invasion chambers (San Jose, CA, USA) according to the manufacturer’s instruction. Images were captured by light photomicrography and quantitation was performed using Image J software V1.47 (Rasband, W.S., ImageJ, U. S. National Institutes of Health (Bethesda, MD, USA, http://imagej.nih.gov/ij/, 1997–2012). For proliferation assays, cells were plated in triplicate at a density of 5 × 10^4^ cells/35-mm dish in defined medium. HGF ± crizotinib or cabozantinib were added on days 1, 2, and 4. Cells were trypsinized and counted using a hemocytometer on days 3 or 6.

### 4.5. Anchorage-Independent Colony Formation Assay

A base layer of 0.5% Noble agarose (Difco, Franklin Lakes, NJ, USA) in phenol-red free DMEM was added to 96 well plates. Cells in 0.3% agarose in phenol-red free DMEM were added on top of the base layer. Cells were fed with DMEM with or without HGF and crizotinib or cabozantinib every other day. After one week, MTT was added to quantify the viable colonies by absorbance using a Victor plate reader (PerkinElmer, Hopkinton, MA, USA).

### 4.6. Tumor Xenograft Models

Experiments with SCID and hHGF/SCID mice were performed in accordance with National Institutes of Health Guidelines for Care and Use of Laboratory Animals using protocols approved by the Institutional Animal Care and Use Committee of the Center for Cancer Research, National Cancer Institute. hHGF/SCID mice (HGF/SCID: Rideout WM 3rd Mouse Genome Database (MGD) at the Mouse Genome Informatics website, The Jackson Laboratory (Bar Harbor, MN, USA; http://www.informatics.jax.org) were acquired from The Jackson Laboratory. SW780 cells (3 × 10^6^ per site) suspended in matrigel were injected subcutaneously into both flanks of 5 mice per treatment group, and tumor volumes were calculated from caliper measurements. When tumor volume reached 150 mm^3^, mice were treated with either vehicle or cabozantinib once daily by oral gavage. Tumors excised from mice were disrupted in non-ionic detergent containing buffer, lysates were cleared by centrifugation, and analyzed using two-site immunoassays for Met and pMet [33].

### 4.7. Statistical Analysis

Significant differences between two groups were determined by One Way ANOVA with post hoc comparison of all groups using GraphPad Prism 5 software, where *p* = 0.05 was the threshold for statistical significance.

## 5. Conclusions

Met protein content in 12 UC-derived cell lines ranged from 12 ng/mg to 133 ng/mg total protein, comparable to that of several other well-studied tumor-derived cell lines from other prevalent cancers. HGF production was conspicuously absent, but exogenous HGF stimulated the activation of Met and known downstream effectors, and significantly enhanced UC cell invasion, proliferation and anchorage-independent growth. Concomitant treatment with cabozantinib, crizotinib or EMD1214063 effectively suppressed these HGF-driven effects. Cabozantinib also significantly inhibited HGF-driven tumor xenograft growth and Met activation in SCID and, more dramatically, in hHGF/SCID mice. Our results suggest that further studies are warranted to fully define the prevalence and oncogenic impact of HGF/Met signaling in bladder cancer, and to identify reliable and practical biomarkers of pathway activation for patient selection and predicting response to treatment with HGF/Met targeted therapeutics.

## References

[1] Siegel R., Naishadham D., Jemal A. (2012). Cancer statistics. CA Cancer J. Clin..

[2] Stein J.P., Lieskovsky G., Cote R., Groshen S., Feng A., Boyd S., Skinner E., Bochner B., Thangathurai D., Mikhail M. (2001). Radical cystectomy in the treatment of invasive bladder cancer: Long-term results in 1054 patients. J. Clin. Oncol..

[3] Von der Maase H., Sengelov L., Roberts J.T., Ricci S., Dogliotti L., Oliver T., Moore M.J., Zimmermann A., Arning M. (2005). Long-term survival results of a randomized trial comparing gemcitabine plus cisplatin, with methotrexate, vinblastine, doxorubicin, plus cisplatin in patients with bladder cancer. J. Clin. Oncol..

[4] Botteman M.F., Pashos C.L., Redaelli A., Laskin B., Hauser R. (2003). The health economics of bladder cancer: A comprehensive review of the published literature. Pharmacoeconomics.

[5] Cecchi F., Rabe D.C., Bottaro D.P. (2012). Targeting the HGF/Met signaling pathway in cancer therapy. Expert. Opin. Ther. Targets.

[6] Yamamoto N., Mammadova G., Song R.X., Fukami Y., Sato K. (2006). Tyrosine phosphorylation of p145met mediated by EGFR and Src is required for serum-independent survival of human bladder carcinoma cells. J. Cell Sci..

[7] Tamatani T., Hattori K., Iyer A., Tamatani K., Oyasu R. (1999). Hepatocyte growth factor is an invasion/migration factor of rat urothelial carcinoma cells *in vitro*. Carcinogenesis.

[8] Inui M., Nishi N., Yasumoto A., Takenaka I., Miyanaka H., Matsumoto K., Nakamura T., Wada F. (1996). Enhanced gene expression of transforming growth factor-alpha and c-met in rat urinary bladder cancer. Urol. Res..

[9] Sanchez-Carbayo M., Socci N.D., Lozano J.J., Haab B.B., Cordon-Cardo C. (2006). Profiling bladder cancer using targeted antibody arrays. Am. J. Pathol..

[10] Cheng H.L., Trink B., Tzai T.S., Liu H.-S., Chan S.-H., Ho C.-L., Sidransky D., Chow N.-H. (2002). Overexpression of c-met as a prognostic indicator for transitional cell carcinoma of the urinary bladder: A comparison with p53 nuclear accumulation. J. Clin. Oncol..

[11] Miyata Y., Sagara Y., Kanda S., Hayashi T., Kanetake H. (2009). Phosphorylated hepatocyte growth factor receptor/c-Met is associated with tumor growth and prognosis in patients with bladder cancer: Correlation with matrix metalloproteinase-2 and -7 and E-cadherin. Hum. Pathol..

[12] Iyer G., Al-Ahmadie H., Schultz N., Hanrahan A.J., Ostrovnaya I., Balar A.V., Kim P.H., Lin O., Weinhold N., Sander C. (2013). Prevalence and co-occurrence of actionable genomic alterations in high-grade bladder cancer. J. Clin. Oncol..

[13] Cerami E., Gao J., Dogrusoz U., Gross B.E., Sumer S.O., Aksoy B.A., Jacobsen A., Byrne C.J., Heuer M.L., Larsson E. (2012). The cBio cancer genomics portal: An open platform for exploring multidimensional cancer genomics data. Cancer Discov..

[14] Gao J., Aksoy B.A., Dogrusoz U., Dresdner G., Gross B., Sumer S.O., Sun Y., Jacobsen A., Sinha R., Larsson E. (2013). Integrative analysis of complex cancer genomics and clinical profiles using the cBioPortal. Sci. Signal.

[15] Guo G., Sun X., Chen C., Wu S., Huang P., Li Z., Dean M., Huang Y., Jia W., Zhou Q. (2013). Whole-genome and whole-exome sequencing of bladder cancer identifies frequent alterations in genes involved in sister chromatid cohesion and segregation. Nat. Genet..

[16] The Cancer Genome Atlas Research Network (2014). Comprehensive molecular characterization of urothelial bladder carcinoma. Nature.

[17] Experimental Cancer Therapeutics Targeting the Hepatocyte Growth Factor/Met Signaling Pathway. https://ccrod.cancer.gov/confluence/display/CCRHGF/Home.

[18] Xu H., Stabile L.P., Gubish C.T., Gooding W.E., Grandis J.R., Siegfried J.M. (2011). Dual blockade of EGFR and c-Met abrogates redundant signaling and proliferation in head and neck carcinoma cells. Clin. Cancer Res..

[19] Yamazaki S., Skaptason J., Romero D., Lee J.H., Zou H.Y., Christensen J.G., Koup J.R., Smith J.B., Koudriakova T. (2008). Pharmacokinetic-pharmacodynamic modeling of biomarker response and tumor growth inhibition to an orally available cMet kinase inhibitor in human tumor xenograft mouse models. Drug Metab. Dispos..

[20] Zou H., Li Q., Lee J.H., Arango M.E., McDonnell S.R., Yamazaki S., Koudriakova T.B., Alton G., Cui J.J., Kung P. (2007). An orally available small-molecule inhibitor of c-Met, PF-2341066, exhibits cytoreductive antitumor efficacy through antiproliferative and antiangiogenic mechanisms. Cancer Res..

[21] Yakes F.M., Chen J., Tan J., Yamaguchi K., Shi Y., Yu P., Qian F., Chu F., Bentzien F., Cancilla B. (2011). Cabozantinib (XL184), a novel MET and VEGFR2 inhibitor, simultaneously suppresses metastasis, angiogenesis, and tumor growth. Mol. Cancer Ther..

[22] You W.K., Sennino B., Williamson C.W., Falcón B., Hashizume H., Yao L., Aftab D.T., McDonald D.M. (2011). VEGF and c-Met blockade amplify angiogenesis inhibition in pancreatic islet cancer. Cancer Res..

[23] Sennino B., Ishiguro-Oonuma T., Wei Y., Naylor R.M., Williamson C.W., Bhagwandin V., Tabruyn S.P., You W., Chapman H.A., Christensen J.G. (2012). Suppression of tumor invasion and metastasis by concurrent inhibition of c-Met and VEGF signaling in pancreatic neuroendocrine tumors. Cancer Discov..

[24] Nguyen H.M., Ruppender N., Zhang X., Brown L.G., Gross T.S., Morrissey C., Gulati R., Vessella R.L., Schimmoller F., Aftab D.T. (2013). Cabozantinib inhibits growth of androgen-sensitive and castration-resistant prostate cancer and affects bone remodeling. PLoS One.

[25] Bladt F., Faden B., Friese-Hamim M., Knuehl C., Wilm C., Fittschen C., Grädler U., Meyring M., Dorsch D., Jaehrling F. (2013). EMD 1214063 and EMD 1204831 constitute a new class of potent and highly selective c-Met inhibitors. Clin. Cancer Res..

[26] McNeil B., Sorbellini M., Athauda G., Apolo A.B., Cecchi F., Athauda G., Cohen B., Giubellino A., Simpson H., Agarwal P.K. (2014). Preliminary evaluation of urinary soluble Met as a biomarker for urothelial carcinoma of the bladder. J. Trans. Med..

[27] Coxon A., Rex K., Meyer S., Sun J., Sun J., Chen Q., Radinsky R., Kendall R., Burgess T.L. (2009). Soluble c-Met receptors inhibit phosphorylation of c-Met and growth of hepatocyte growth factor: C-Met-dependent tumors in animal models. Mol. Cancer Ther..

[28] Li B., Kanamaru H., Noriki S., Fukuda M., Okada K. (1998). Differential expression of hepatocyte growth factor in papillary and nodular tumors of the bladder. Int. J. Urol..

[29] Wang P., Nishitani M.A., Tanimoto S., Kishimoto T., Fukumori T., Takahashi M., Kanayama H. (2007). Bladder cancer cell invasion is enhanced by cross-talk with fibroblasts through hepatocyte growth factor. Urology.

[30] Rosen E.M., Joseph A., Jin L., Yao Y., Chau M.T., Fuchs A., Gomella L., Hastings H., Goldberg I.D., Weiss G.H. (1997). Urinary and tissue levels of scatter factor in transitional cell carcinoma of bladder. J. Urol..

[31] Chiba S., Tsuchiya N., Horikawa Y., Narita S., Inoue T., Akihama S., Saito M., Numakura K., Tsuruta H., Huang M. (2014). Functional mononucleotide repeat polymorphism in the promoter region of HGF is associated with risk and malignant aggressiveness of bladder cancer. Int. J. Oncol..

[32] Apolo A.B., Lee Y.H., Cecchi F., Agrawal P.K., Parnes H., Khadar K., Summerell A., Gulley J.L., Compton K., Figg W.D. (2013). Preclinical and correlative studies of cabozantinib (XL184) in urothelial cancer (UC). J. Clin. Oncol..

[33] Athauda G., Giubellino A., Coleman J.A., Horak C., Steeg P.S., Lee M., Trepel J., Wimberly J., Sun J., Coxon A. (2006). c-Met ectodomain shedding rate correlates with malignant potential. Clin. Cancer Res..

